# “I don’t go to funerals anymore”: how people who use opioids grieve drug-related death in the US overdose epidemic

**DOI:** 10.1186/s12954-022-00693-7

**Published:** 2022-10-01

**Authors:** Allison V. Schlosser, Lee D. Hoffer

**Affiliations:** 1 Sociology and Anthropology Department, University of Nebraska Omaha, Arts and Sciences Hall Room 383N, 6001 Dodge Street, Omaha, NE 68182 USA; 2 Anthropology Department, Case Western Reserve University, Mather Memorial Building Room 211, 11220 Bellflower Road, Cleveland, OH 44106 USA

**Keywords:** Opioid use, Drug overdose death, Bereavement, Complex grief, Naloxone, Qualitative research

## Abstract

**Background:**

Opioid-related overdose death is a public health epidemic in much of the USA, yet little is known about how people who use opioids (PWUO) experience overdose deaths in their social networks. We explore these experiences through a qualitative study of opioid-related overdose death bereavement among PWUO.

**Methods:**

We recruited 30 adults who inject opioids from a syringe service program in the Midwestern USA and interviewed them using a semi-structured guide that addressed experiences of opioid use, opioid-related overdose, and overdose reversal via the medication naloxone. Interviews were transcribed verbatim and analyzed thematically.

**Findings:**

Participants described overdose death as ever-present in their social worlds. Most (approximately 75%) reported at least one overdose death in their social network, and many came to consider death an inevitable end of opioid use. Participants described grief shaped by complex social relations and mourning that was interrupted due to involvement with social services and criminal legal systems. They also reported several ways that overdose deaths influenced their drug use, with some increasing their use and others adopting safer drug use practices. Despite the high prevalence of overdose deaths in their social networks, only one participant reported receiving grief support services.

**Discussion:**

Findings underscore the need for interventions that not only maintain life, such as naloxone distribution, but also improve quality of life by attending to grief related to overdose death bereavement. We discuss policies and practices with the potential to address the unique psychological, social, and structural challenges of grief for this population.

## Introduction

The USA is struggling with a drug overdose death epidemic. Since 1999, 932,000 people have died from drug overdose in the USA. In 2020, there were 91,799 drug overdose deaths, with 75% involving an opioid [1]. The age-adjusted rate of overdose deaths from 2019 to 2020 increased from 21.6 to 28.3 deaths per 100,000 [1]. Provisional data suggest that over 100,000 drug overdose deaths occurred in the 12 months ending in April 2021; an increase of almost 30% compared to the prior year [2]. The current wave of the drug overdose epidemic is driven by deaths related to synthetic opioids such as fentanyl and its analogs [3].

The drug overdose epidemic is resulting in a growing number of people bereaved by drug overdose death. Yet the experiences of people bereaved by drug overdose death—an understudied and stigmatized social group—are poorly understood [4–7]. This paper explores experiences of bereavement among people who use opioids (PWUO) who have lost a member of their social network—family members, friends, and associates that individuals identify as important in their lives—to a drug overdose. Social networks are important sources of social and emotional support [8], and studies demonstrate that they are a significant resource of support for people who use drugs [9, 10]. Drawing on qualitative interviews with PWUO bereaved by drug overdose death, we aim to add depth and nuance to our understanding of this largely unstudied, but increasingly common, experience.

### Social context and stigma

Grief, as Dyregrov et al. stress, “rarely arises in a social vacuum” [4, p 416]. Rather, it is inextricably connected to social context. Thus, scholars have stressed the need to attend to the social contexts of grief. Rosenblatt, for example, emphasizes the importance of others in shaping how a person defines, feels, and comes to terms with a loss [11]. Scholars have also highlighted the social regulation of grief, noting that grief may not be considered a socially legitimate right in cases of stigmatized relationships and/or the loss of others who are socially devalued [12]. In the case of drug-related death (DRD), the social contexts of bereavement often challenge the grieving process. Research on the social contexts of DRD bereavement highlights the ways in which social stigma affects people bereaved by DRD, particularly family members of the deceased [4–7]. In general, social support is critical for healthy grieving [13]. The ability for the bereaved to be open about the loss and their needs for support is particularly important for healthy grieving [4]. Social stigma associated with DRD, however, may hinder the openness that fosters social support.

The concept of social stigma has been expanded from Erving Goffman’s early framing of it as a mark that leads to “spoiled identity” to a process in which people with a certain social identity are labeled, stereotyped, and devalued [14–16]. Importantly, these processes unfold in contexts marked by unequal power relations, which results in discrimination against people with a stigmatized identity [14, 16]. Research on the experiences of people bereaved by DRD reflects these stigma processes. Dyregrov and Selseng [5], for example, found that people bereaved by DRD were subject to derogatory comments about the death from members of their social networks, including close and/or extended family and friends, work colleagues, neighbors, and even professionals. Comments included dehumanizing labeling, implying stigma, blaming the deceased, and referring to death as the only and best outcome.

Moreover, people who use drugs—and, by association, their family members—are often viewed as complicit in, or at fault for, the death [17–19]. These dynamics can result in shame and self-stigma among people bereaved by drug overdose death [20, 21]. Self-stigma occurs when a person with a stigmatized identity becomes aware of the stigma, agrees with it, and applies it to themselves, negatively impacting their self-esteem and self-efficacy [14, 20]. Compounding these problems, there are increasing trends in PWUO being charged with “drug-induced homicide” as a consequence of their drug use with the deceased [22–24]. As a result, people bereaved by DRD may suffer from feelings that they have failed the deceased [18], “associative stigma” due to their connection to the deceased [5], tense relationships with others [7], and social isolation related to the bereaved person’s perceived need to conceal the DRD [25].

Additionally, people bereaved by DRD must contend with structural stigma: “societal-level conditions, cultural norms, and institutional practices that constrain the opportunities, resources, and well-being for stigmatized populations.” [26, p 2] Structural stigma affects a wide range of societal institutions with which people bereaved by DRD frequently engage while grieving, including healthcare, social services, and criminal legal systems. Interactions with these institutions may exacerbate the stress of DRD bereavement. Templeton et al. [7] for example, found that DRD bereaved persons’ encounters with health and social services involved navigating unknown and fragmented systems that added to their distress. Structural stigma may be intentional, such as policies or practices that limit or deny access to services for individuals, or inadvertent, such as when limited access to services is due to funding constraints [27]. Inadvertent structural stigma, however, is more difficult to identify [27]. Attention to structural stigma highlights the need to examine how experiences of grieving DRD are shaped by a country’s policies related to illegal drug use that may exacerbate stigma and intensify distress, such as drug criminalization [4].

The self, social, and structural stigma associated with DRD may lead to disenfranchised grief: that is, grief that is unacknowledged by one’s social network and/or by society more generally [28].

### Complex grief

Scholars have described DRD—along with death by suicide, AIDS, and murder—as especially challenging due to the circumstances of the death [6, 7, 19]. These deaths are often marked by significant trauma, social stigma, and existential pain, with the ensuing grief commonly disenfranchised [6]. They are also associated with increased rates of post-traumatic stress disorder, major depressive disorder, and the development of complex grief [7, 21, 29, 30]. Complex grief is marked by feelings of debilitating loss that persist, resulting in an ongoing, heightened state of grief that may hinder one’s ability to accept the reality of the loss, feel the pain of the loss, adjust to life without the deceased, and foster new relationships [31].

PWUO may be particularly at risk for complex grief. Opioid-related drug overdose deaths are often socially constituted as “ungrievable”—less worthy of grief and memorialization compared to other avoidable deaths—reflecting the significant stigma associated with opioid use [32, 33]. The social stigma of opioid-related overdose death may be compounded when the bereaved is themselves a person who uses opioids. Members of the social networks of PWUO, who may also use opioids, are most likely to be the first responders in the event of an overdose [34, 35]. Moreover, people who struggle with substance use have a significantly increased risk of complicated grief reactions [36].

While scholars have called for greater attention to the needs of people who use drugs bereaved by DRD [7], we know little about their experiences. With these research gaps in mind, we explore how PWUO experience drug overdose death bereavement based on a qualitative interview study conducted in the Midwestern USA in 2019.

## Methods

This article is based on a 2019 National Drug Early Warning System (NDEWS) “HotSpot” study of opioid use in Cleveland, Ohio. NDEWS HotSpot studies are funded by the USA National Institute on Drug Abuse (NIDA) and are designed to explore emerging drug problems and provide findings that inform policy and practice. Cleveland, a mid-sized city in the Midwestern USA, was identified as a HotSpot study site because fentanyl—a potent opioid that significantly increases the risk of overdose when it is taken—is endemic in the local illegal drug market [37]. Our approach implemented a hybrid of rapid qualitative inquiry (RQI) [38] and rapid ethnographic assessment (REA) [39] in conducting qualitative interviews to explore beliefs and practices related to opioid use, the presence of fentanyl in the local illegal drug market, opioid-related overdose, and the use of take-home naloxone to prevent overdose death [40]. Both RQI and REA are systematic approaches to rapidly collect primary and secondary data, in collaboration with community members, to address targeted topics. Because this study included in-depth qualitative interviews designed to capture depth, nuance, and context in participant experiences but did not include fieldwork, it did not meet the full criteria for REA. The topic of DRD bereavement was not the initial focus of the study, but it quickly emerged as a prominent topic.

### Sampling and recruitment

We collaborated with a local Syringe Services Program (SSP), which was connected to an overdose education and naloxone distribution (OEND) program, to recruit participants using an opportunistic and purposive sampling approach. Due to its targeted and time-limited focus, it is important in REA to recruit a sample that is, “especially knowledgeable about or experienced with a phenomenon of interest.” [39, p 50] We also wanted to recruit a sample that included frequent and less regular users. Individuals were eligible to participate if they were: adults (age 18 or older), clients of the SSP, not in residential treatment, reported having injected opioids at least five times in the last 30 days, and not had been interviewed previously for this study.

SSP staff informed clients of the opportunity to participate in the study after they completed their SSP visit. If the client expressed interest, the SSP staff person introduced them to a study researcher. The researcher then described the study and obtained verbal consent for participation in a private room in the free health clinic where the SSP is located. Verbal consent was obtained instead of written consent to protect the participant’s identity by eliminating the requirement for them to sign a consent document, which increases the risk of breach of confidentiality. A one-time confidential interview was then conducted in the private clinic room. Individuals received 20 USD compensation for their participation. All interviews were audio-recorded and transcribed verbatim. All names that appear in this article are pseudonyms to protect participant confidentiality. This study received ethical approval from the Case Western Reserve University Institutional Review Board (IRB # 20180240). There were no study protocol deviations.

### Interviews

We conducted 30 open-ended, semi-structured interviews with people who inject opioids in February and March 2019. Interviews lasted from 40 to 70 min (average 47 min). Similar to other HotSpot studies, the interview guide developed for this study was designed to interrogate recent trends in drug use. After collecting socio-demographic information, interviews focused on drug use histories, current opioid use patterns, local drug market dynamics, responses to fentanyl in the local drug supply, and beliefs and practices related to naloxone. Our sample had high rates of exposure to fatal overdose, and questioning participants about naloxone unexpectedly generated detailed personal narratives about these experiences within their social networks. This article focuses on these narratives as bereavement related to drug overdose was a topic that quickly emerged in response to a single interview question followed by several probes about opioid-related overdose death in the participant’s social network.

Our research team prepared for the possibility that these questions could trigger emotional distress among participants by making referrals to local mental health services available. We debriefed with participants after their interview was complete, asking them what it was like for them to participate in the interview and offering to connect them with mental health services. Our research team also discussed participant narratives about overdose death and grief, which assisted us in processing this emotionally charged data. Similar to other studies using in-depth interviewing approaches, participants often told us they appreciated the opportunity to discuss their experiences with DRD bereavement during the interview and debriefing, but none requested a mental health service referral.

### Analysis

We use an iterative, team-based thematic analytic approach informed by RQI [38]. RQI allowed the research team to quickly attain a sufficient understanding of findings related to a largely unexplored topic of opioid use in a fentanyl hotspot. Although the interview guide included questions about fentanyl in the local drug market and the use of naloxone, the findings reported below about processing grief from overdose death experiences were not targeted as a topic. The challenges with grief emerged in the interviews and analysis. Three researchers conducted interviews and analyses. After each interview, the interviewer took notes on prominent topics and early observations from the interview. Between interviews, they re-listened to interview audio to identify and elaborate a priori and emergent themes that informed future interviews. DRD bereavement emerged as a prominent topic that the researchers noted after the first few interviews. The interview guide already included a stem question about overdose deaths in the participant’s social network but following our discussions of the prominence of this topic in early interviews, we noted the need to follow up with probes aimed at better understanding these experiences (e.g., “How did that make you feel?”).

Interview notes and transcripts were organized using QSR Nvivo 12 qualitative data analysis software. The data were analyzed in stages. The first author (AVS) identified broad categories in the data based on familiarization with all transcripts and notes, resulting in the creation of the initial codebook following a “first cycle coding” process [41]. All study team members then reviewed the data and codebook and discussed themes and patterns in the data, resulting in the addition of new codes, edits to codes, and the collapsing of codes. The final codebook was then used by AVS to code all data in a process of “second cycle coding” [41].

### Sample

We achieved thematic saturation with a sample size of 30, which was enabled by the focused nature of the study [42]. Our final sample largely identified as male (76%) and White non-Hispanic (83%). Only 10% of the sample identified as African American and 2% as Hispanic. Participants ranged in age from 20 to 68, with an average age of 39.

## Findings

Our analyses reveal four themes that illustrate how PWUO in our sample experience opioid-related overdose death bereavement: (1) social worlds saturated with death; (2) conflicts in social relationships; (3) interrupted mourning; and (4) drug use in the wake of overdose death. In the following sections, we present particularly information-rich cases to illustrate these themes [43].

### “That’s the world I’m in”: social worlds saturated with death

Most participants—about 75%—reported at least one opioid-related overdose death in their social network in their lifetime. They described overdose death as ubiquitous in their social worlds as they lost family members, close friends, and drug market “associates” to an overdose death. Mike, a White man in his late 30s, traced the names etched onto his arm as he discussed the “lifelong friends” that he lost to overdose death. “I’ve got them tattooed on my body,” he said as he looked down at the names, “some of my best friends.”

Some participants described losing multiple friends and family members in a brief time—sometimes a matter of weeks—leading to them becoming desensitized to overdose death. Matt, a White man in his late 30s, stressed the pervasiveness of overdose death in his social network: “The amount of people that are dying, it’s not a drug anymore. It’s like you’re buying a casket.” He went on to explain:

I don’t go to funerals anymore, because everybody I know, you know.... [pause] My life’s been in and out of recovery since I was like 13. So once the epidemic hit, everybody knew somebody, you know? And then, you’re going to funerals. And it was people dying too soon and all that. And *now it’s happening so regularly that it doesn’t even faze m*e. It’s still sad, but like, you know—yeah. Yeah, I just had two or three friends die in the last couple of weeks. [Emphasis added]

Others believed that DRD is inevitable because of the large number of overdose deaths in their social networks despite their own experiences of surviving an overdose. Participants, like Jason, a White man in his early 30s, described a profound sense of fatalism about his own potential drug overdose death:

I lost my brother to it. Yeah, that’s the world I’m in.... [pause] I lost my aunt to carfentanil [a potent opioid]—she overdosed last August. It was only a few weeks after I overdosed, so it was kind of crazy. I’ve already lost two people that were close to me, especially my brother. Really close to me. I lost a lot of good friends. If you’re in that world, you know that it’s gonna cost you your life eventually. You kind of just know, and your life sucks so much anyways that you’re just kind of like, *if it happens, it happens*. It ends this chaos. [Emphasis added]

Jake similarly expressed fatalism related to overdose death; in his case, in relation to his girlfriend who ultimately died of a fatal opioid-related overdose:

It’s not a cool feeling [after you reverse an overdose with naloxone]. Once it happened more than once, especially with my girlfriend, I was like, ‘Well, when I’m not there, she’s gonna die. If Narcan is not there, she’s gonna die.’ I didn’t see her not using [opioids], and that’s eventually what happened. She used when no one was around, and she died. So, I kind of like accepted the fatalness of it.

Despite the commonly expressed fatalism associated with opioid-related overdose death, most participants at the same time described their fear of death. Throughout her interview, Justine (White woman, mid-30s) repeated: “I’m afraid of death. I don’t wanna die.” For participants like Justine, the inevitability of drug overdose death existed in tension with their intense fear of death.

### “They thought I was responsible for it”: conflicts in social relationships

In addition to living in social worlds saturated with death, participants described conflict in their social relationships after a drug overdose death in their social network—particularly in cases of the death of a close friend or family member. Social conflict was often connected to the belief among others close to the deceased that the participant was responsible for the death. Armando, a Hispanic man in his early 20s, described social tension after the drug overdose death of a particularly close friend:

Armando: I knew a lot of people in high school [who died of a drug overdose]. I knew a couple of different people—like I *knew* them. My one friend that I grew up with, Chad, he overdosed and died.

Interviewer: How did that affect you?

Armando: It made me feel guilty, because I’m not gonna say I didn’t start it. He obviously did shit on his own, but we—he was my friend and we got high together. I just feel like we went to the same place together. I wasn’t.... we split off, stopped, he started doing his own thing, hanging out with different people, getting high and shit. And I don’t know. His whole family—they thought I was responsible for it. Like I sold him the dope or this and that. I went to the funeral and shit, and it was a fucked-up situation.

Interviewer: Did it influence your drug use at all?

Armando: Initially, it made me get high more, for real. It kind of put me in a depression. I just kept getting high.

The social conflicts and emotions that Armando experienced—particularly accusations of fault for the overdose death and feelings of guilt for having used drugs with the deceased—were common among participants and lead some to isolate themselves socially and/or experience intensified suffering. While Armando attended his friend Chad’s funeral—which ended up being a “fucked up” conflict-filled situation—other participants intentionally did not attend funerals to avoid social conflict, stripping them of the opportunity to grieve with others through this central mourning ritual.

### “I never got to say bye”: interrupted mourning

Missing funerals to avoid social conflict is one example of several ways in which participants’ mourning was interrupted. Participant experiences of interrupted mourning were also shaped by their involvement with criminal legal and social services systems. Amanda (White woman, early 40s), for example, lost her brother, with whom she was close, to drug overdose death while she was incarcerated on a drug-related charge. She was unable to attend his funeral because she was incarcerated, and she struggled to process the death. “Because I never got to say bye to him,” Amanda explained, “it’s really affected me.” She thought about her brother often in the year following his death and attempted to keep him alive in her mind. “I talk to him,” she said, “He watches over me.”

Despite the high prevalence of drug overdose deaths in their social networks, Amanda was the only participant in our sample who reported receiving grief support services. She was living in a women-only “sober living” facility up until a week before her interview and received grief support counseling there. Yet she was discharged against her wishes after she visited a friend who was not approved by the program while she was off-site. This program rule violation—unrelated to drug use—led her to lose not only her access to safe housing, but also to grief counseling:

Amanda: I was in grief and loss counseling in sober living, so everything’s kind of dropped off right now.

Interviewer: What was the counseling like?

Amanda: It was good. It has you do like these pamphlets about what you would say to him now. What he missed this year. He was really close to my daughter, and she just had a baby, so, you know. His daughter is pregnant right now, and was just getting things out. I could still talk to him. Then, things I’m mad at him for. It’s a really good process. It helps you to let go and not feel the guilt of letting go. They’re gone, so you gotta move on with your life. But that don’t mean you have to forget them. That’s what I held onto. I felt like if I let him go, then that was wrong of me. But it’s not, as long as I don’t forget him.

Amanda found the grief counseling offered at the facility helpful and planned to return as soon as possible but would have to wait 30 days before she could re-apply for admission. The institutional contexts of Amanda’s grieving—prison and supported housing—interrupted her morning for over a year after the death of her brother. Ironically, the grief support that Amanda received to help her process her interrupted mourning was itself interrupted by her discharge from the program due to its rules restricting social contact.

### Drug use in the wake of overdose death

Participants reported several ways in which drug overdose deaths in their social networks influenced their drug use. A common response to their grief was to increase their drug use. Jason, whose fatalistic attitude toward opioid use after the death of his brother was described above, increased his heroin use to numb the pain of his grief:

Now I’m using about a gram, gram-and-a-half a day. There was a time when I was using about three or four after my brother died. I was trying to just use until it was over and I didn’t wake up, but that didn’t happen, so eventually I got sober again, and then when I went back to it.

Rick, a White man in his late 40s, reported 15–20 opioid-related overdose deaths in his social network, including his immediate family members. When asked how that influenced his life and drug use, he responded:

I guess the drug use has actually increased to suppress pain and feelings you have. There’s so much regret and, you know.... I mean, it sucks. My family’s pretty much gone. You know, I grew up with two brothers and I was real close with my mom, and they’re all—you know, my mom was a drug addict, too, so—she just died last year.

Less common, participants responded to overdose deaths by reducing their drug use and adopting harm reduction practices. For example, Amanda, who experienced interrupted mourning after the drug overdose death of her brother, adopted safer drug use practices due to her fear that she might also die from a drug overdose:

If I do it [opioids], it’s very little, because people are just dyin’ like crazy off of it. It scares me. My brother OD’d [overdosed] and died a week before I got out of prison.... I’m scared. I ain't even gonna lie. I’m scared.

Later, she explained that she is more intentional about using harm reduction practices in her drug use since her brother’s DRD because, “I don’t ever wanna make my mom feel like I felt” after the death of her brother.

Most often, participants reported shifting between eliminating or limiting drug use and increasing their drug use. Matt, whose experience of pervasive drug overdose death in his social network was described above, recounted the drug overdose death of a close friend. After using opioids together, Matt thought his friend was falling asleep in his chair and carried him to bed. In the morning, he found him dead of an apparent opioid-related overdose:

Matt: It’s a scary—I mean, I put one of my friends to bed and, you know—so my friend was overdosing. And I didn’t know it. I thought he was just like sleeping at the table. So I tipped him back in his chair and I dragged him to his bed and put him to bed. And then in the morning, I woke up and he was dead.

Interviewer: Oh, wow.

Matt: And if I would’ve had naloxone, if I would’ve known—I mean, this was a while ago—then I would’ve reacted a lot differently, but it was one of those situations that I just—yeah.

Interviewer: When did this happen?

Matt: Probably six years ago.

Interviewer: How did that make you feel?

Matt: I breathed out of a paper bag for like a week and I had panic attacks and all that. And I thought it would be enough for me to like, ‘Okay, this is it. I gotta get my shit together,’ and whatever. And it was for a year and a half. It was enough for me to kind of think about it every day and use it to benefit, but it wasn’t—it wasn’t enough to keep me sober.

Matt did not receive any grief support services and continued to struggle with panic attacks and anxiety long after the death of his friend. When asked for final comments at the close of his interview, Matt noted that no one has ever asked him how the death of his friend made him feel. When he obtained naloxone at the OEND program that was connected to the SSP, the staff asked him a number of times that he used naloxone and the outcome, but not his reactions to these experiences.

## Discussion

This article describes experiences of DRD bereavement among PWUO, which were characterized by living in social worlds saturated with death, contending with conflicts in social relationships, and suffering from interrupted mourning. In response, study participants altered their drug use in the wake of a drug overdose death in their social network, with most toggling between periods of intensified drug use to numb the pain of their grief to abstinence or safer drug use practices to protect themselves from death. Our findings echo research showing that PWUO often develop a sense that overdose death is an inevitable result of opioid use, leading to fatalism and ambivalence to overdose that may diminish their willingness to engage with harm reduction services and practices, especially among those who are structurally vulnerable [35, 44–46]. Complex grief in DRD bereavement may be a factor in these more general findings about fatalism and ambivalence to overdose among PWUO, although it is not often discussed in the literature. Participants in this study lived in and through this sense of death’s inevitability as they grappled with self-, social, and structural stigma. These findings give voice to people whose bereavement has been largely absent from research and whose grief reactions are poorly understood. In attending to these experiences, we join scholars who aim to break the “sounds of silence” [6] associated with DRD [4, 5, 7].

### Multi-level stigma

Stigma shaped the drug overdose death bereavement of PWUO on multiple intertwined levels: self-, social, and structural. Participants described feelings of guilt that they were responsible for the opioid-related overdose death due to their history of using drugs with the person, especially when it was a close friend, which was most often the case. This guilt was sometimes exacerbated by the responses of others in the person’s social network, particularly the family members and friends of the deceased. These dynamics reflect the compounded stigma of being a DRD bereaved person who is also a member of the highly stigmatized group of PWUO. Self- and social stigma often intersected to lead participants to isolate themselves socially, in particular, to avoid the funerals of the deceased, interrupting their grieving process.

Interrupted mourning was also influenced by structural stigma in intentional and inadvertent ways. As other research on DRD bereavement found, the participants in this study got little-to-no-grief support from formal health or social services [6]. This near-total lack of grief support services likely reflects intentional structural stigma. Participants’ involvement with criminal legal and social services systems also resulted in interrupted mourning that could increase the likelihood of them developing complex grief. Incarceration and institutionalization limited some participants’ ability to attend funerals and access social and psychological support for grieving. Other punitive institutional policies—such as those of Amanda’s housing program—lead to inadvertent structural stigma by interrupting her access to grief counseling. These forms of structural stigma underscore the need to critically examine the ways in which the criminalization of drug use and other punitive policies directed at people who use drugs leads to disenfranchised grief and prolonged complicated grief reactions. The decriminalization of drug use and possession for personal use has the potential to reduce the stigma associated with drug use by undoing the conflation of drug use with criminal behavior [14]. It may also promote help-seeking among people who use drugs by reducing their fear of criminal sanctions [47, 48]. While these policy changes alone will not guarantee access to grief support for people who use drugs, they are important steps in addressing structural impediments to healthy grieving among PWUO.

### Implications: beyond survival

The participants in this study were engaged with harm reduction services: all were SSP clients and accessed take-home naloxone via the associated OEND program. These programs are essential and effective at saving lives and reducing harm to individuals and communities. However, Kolla and Strike [49] note that these types of programs have formed in response to a crisis and exist with a broader policy and legal environment rooted in the criminalization of drug use. This lineage of criminalization will continue to handicap the public health response to drug use. Critical scholars have drawn on a biopolitical perspective to examine the ways in which interventions that aim to address problematic opioid use reduce these individuals to their physiology by focusing on risk reduction and the avoidance of death [50–54]. Bartoszko [50], for example, found that opioid substitution treatment in Norway promotes a “chronic survival modus” by focusing on keeping its clients alive without attention to improving their quality of life. Such interventions often focus on discrete harm reduction practices without attention to the social, affective, and material characteristics of “enabling environments” that support well-being [54].

The DRD bereaved PWUO who we interviewed described a similar phenomenon. Matt, whose close friend died of an opioid-related overdose after he put him to bed, noted that no one asked him how he felt about the death—even the harm reduction services providers with whom he interacted. OEND programs that distribute naloxone routinely ask clients to report the number of people that they have revived with naloxone, but typically do not ask them about how they responded to these experiences. Snoek and Fry describe these approaches as strategies meant to, “protect the biological life by making it the most important thing,” yet they result, “paradoxically in an abandonment of that life.” [53, p 130] This is consistent with the interventions that emerge out of crisis in an “emergency imaginary” [55] in which attention to issues that underlie or exacerbate a crisis is largely ignored and interventions are directed at responding to immediate needs. Notably, harm reduction services often operate in these ways due to limited resources, high demand for services, and funder requirements to measure intervention outcomes based on narrow metrics such as the number of take-home naloxone kits distributed and overdoses reversed.

Beyond keeping PWUO alive and disease-free with SSP and OEND programs, there is a critical need to understand and attend to the grief experienced in response to an overdose death. To this end, we join Dyregrov et al. [4] in calling for systematic and proactive grief support services for DRD bereavement, particularly in highly affected populations of PWUO. Additionally, our findings on the enmeshed dynamics of self-, social, and structural stigma underscore the need for attention to social relations involved in drug overdose death bereavement. Such services should be part of a broader effort in harm reduction to “move beyond a focus on utilitarian individualism and consider the response of social networks to risk as well as […] how risk may mobilize community in the name of mutual protection and healing.” [56, p 29] Changes to criminal legal, healthcare, and housing systems that support the health and well-being of people who use drugs are critical to enabling harm reduction services to carry out these efforts. These structural changes are necessary to give service providers the time and resources to focus on the social and affective needs of PWUD and not only the immediate need to keep them alive.

Mobilizing community in the name of healing is a comprehensive strategy to facilitate grieving among PWUO that is attentive to the unique psychological, social, and structural dynamics of their bereavement. In this way, we might cultivate what anthropologist Angela Garcia calls “death as a resource for life”: an understanding of the ways in which “death deeply penetrates life, not only in the sense of diminishing it, but also in the sense of giving it new resources to survive, perhaps even to flourish” [57, p 318]. To support communal grieving, however, actively diminishing the multi-level stigmas that promote the silence surrounding the experiences of people bereaved by drug overdose death is critical.

Anti-drug war activist groups and peer-led harm reduction organizations, such as Voices of Community Activist and Leaders (VOCAL-NY) and the New England Drug Users Union, are examples of groups that mobilize community to support the health and well-being of people who use drugs [58, 59]. Jarrett Zigon describes such groups as “communities of those without community” [60 p 133]. Importantly, they are organized around “attuned care” characterized by “letting-be” (accepting people unconditionally) and “being-with” (non-judgmental presence and connection) in contrast to the biopolitical “caring-far” that is often focused on normalization and discrete harm reduction practices [60]. These are opportune social spaces in which to support communal grieving. This may take place in support groups such as the recently developed Harm Reduction Works-HRW program [61] or simply in the course of being-with in these communities. Many of these groups have been grieving and memorializing the deaths of people who use drugs for some time. As DRD continues to rise in the USA, greater access to these peer-led communities and support groups is sorely needed.

### Strengths and limitations

While this study provides critical insight into DRD bereavement among PWUO, filling a key gap in the literature, it has limitations. Notably, there were few Black and Latinx participants in this study. Grieving may be experienced differently in these populations, particularly considering the potential for them to be subject to race-based social and structural stigma [62]. Understanding these experiences is especially important as drug overdose deaths have spiked among racialized and BIPOC (Black, Indigenous, and people of color) during the COVID-19 pandemic [63, 64]. Additionally, this study did not examine experiences of DRD bereavement among PWUO in relation to their religious beliefs, educational attainment, employment status, income, or local policies related to drug use and interventions outside of Cleveland, Ohio. Future research should examine these factors as they may influence DRD bereavement among PWUO. Moreover, this study did not involve field observations. Observing how people grieve drug overdose death in their everyday lives would add additional context and nuance to qualitative interview data. Finally, while our findings add depth to our understanding of drug overdose death bereavement among PWUO in the USA, they may not be fully generalizable to this population nor to PWUO in other national contexts due to the focused nature of our research design.

### Conclusion

Our findings fill a critical gap in the literature on how PWUO experience drug overdose death in their social networks. We highlight the unique psychological, social, and structural contexts of these experiences that lead to interrupted mourning, leading to the greater possibility of prolonged suffering through complex grief reactions. Additionally, we underscore the need to understand and address the entangled self-, social, and structural stigmas that these individuals must contend with while grieving. Drug overdose death bereaved PWUO are a largely invisible group, and their grieving has received little research attention. With this work, we aim to begin to break this profound silence and draw attention to the unique experiences and needs of this ever-growing population.

## Data Availability

The datasets generated and analyzed for this study are not publicly available to protect participant confidentiality. The qualitative data collected in this study could be used to identify individuals involved in illegal activities and is therefore only available to the research team.

## Abbreviations

DRD: Drug-related death
NIDA: National Institute on Drug Abuse
NDEWS: National Drug Early Warning System
OEND: Overdose Education and Naloxone Distribution
PWUO: People who use opioids
RQI: Rapid qualitative inquiry
SSP: Syringe services program
USA: United States of America

## References

[1] Centers for Disease Control and Prevention [CDC] [Internet]. Drug overdose deaths remain high. 2020. https://www.cdc.gov/drugoverdose/deaths/ Accessed 20 April 2022.

[2] National Center for Health Statistics [Internet]. Drug overdose deaths in the U.S. top 100,000 annually. 2021. National Center for Health Statistics. Accessed 14 February 2022.

[3] Ciccarone D (2021). The risk of illicit fentanyls, stimulants and the fourth wave of the US opioid overdose crisis. Curr Opin Psychiatry.

[4] Dyregrov K, Møgster B, Løseth H, Lorås L, Titlestad KB (2020). The special grief following drug related deaths. Addict Res Theory.

[5] Dyregrov K, Selseng LB (2022). “Nothing to mourn, he was just a drug addict”: stigma towards people bereaved by drug-related death. Addict Res Theory.

[6] Titlestad KB, Mellingen S, Stroebe M, Dyregrov K (2021). Sounds of silence. The “special grief” of drug-death bereaved parents: a qualitative study. Addict Res Theory..

[7] Templeton L, Ford A, McKell J, Valentine C, Walter T, Velleman R (2016). Bereavement through substance use: findings from an interview study with adults in England and Scotland. Addict Res Theory.

[8] Hall A, Wellman B, Cohen S, Syme S (1985). Social networks and social support. Social support and health.

[9] Kandel DB, Davis M (1991). Friendship networks, intimacy, and illicit drug use in young adulthood: a comparison of two competing theories. Criminology.

[10] Latkin C, Mandell W, Oziemkowska M, Celentano D, Vlahov D, Ensminger M (1995). Using social network analysis to study patterns of drug use among urban drug users at high risk for HIV/AIDS. Drug Alcohol Depend.

[11] Rosenblatt P (1985). Grief: the social context of private feelings. J Soc Issues.

[12] Fowlkes MR (1990). The social regulation of grief. Sociol Forum.

[13] Cacciatore J, Thieleman K, Fretts R, Jackson LB (2021). What is good grief support? Exploring the actors and actions in social support after traumatic grief. PLoS ONE.

[14] Corrigan P, Nieweglowski K (2018). Stigma and the public health agenda for the opioid crisis in America. Int J Drug Policy.

[15] Goffman E (1963). Stigma: notes on the management of spoiled identity.

[16] Link BG, Phelan JC (2001). Conceptualizing stigma. Annu Rev Sociol.

[17] Chapple A, Ziebland S, Hawton K (2015). Taboo and the different death? Perceptions of those bereaved by suicide or other traumatic death. Sociol Health Illn.

[18] Guy P (2004). Bereavement through drug use: messages from research. Practice.

[19] Guy P, Holloway M (2007). Drug-related deaths and the ‘special deaths’ of late modernity. Sociology.

[20] Corrigan P, Watson A, Miller F (2006). Blame, shame, and contamination: the impact of mental illness and drug dependence stigma on family members. J Fam Psychol.

[21] Feigelman W, Jordan J, Gorman B (2011). Parental grief after a child’s drug death compared to other death causes: investigating a greatly neglected bereavement population. Omega.

[22] Carroll JJ, Ostrach B, Wilson L, Dunlap JL, Getty R, Bennett J (2021). Drug induced homicide laws may worsen opioid related harms: an example from rural North Carolina. Int J Drug Policy.

[23] Williams P. The wrong way to fight the opioid crisis. The New Yorker. 2020 Feb. Available from: https://www.newyorker.com/magazine/2020/02/10/the-wrong-way-to-fight-the-opioid-crisis

[24] Goulka J, Beety VE, Kreit A, Boustead A, Newman J, Beletsky L. Drug-induced homicide defense toolkit (2021 edition) (July 30, 2021). Ohio State Public Law Working Paper No. 467. Available from: 10.2139/ssrn.3265510.

[25] Quinn DM, Weisz BM, Lawner EK (2017). Impact of active concealment of stigmatized identities on physical and psychological quality of life. Soc Sci Med.

[26] Hatzenbuehler ML, Link BG (2014). Introduction to the special issue on structural stigma and health. Soc Sci Med.

[27] Livingston JD. Mental illness-related structural stigma: the downward spiral of systemic exclusion. Mental Health Commission of Canada. 2013. Available from: www.mentalhealthcommission.ca.

[28] Doka KJ (2002). Disenfranchised grief: new directions, challenges, and strategies for practice.

[29] Feigelman W, Gorman B, Jordan J (2009). Stigmatization and suicide bereavement. Death Stud.

[30] Kristensen P, Weisaeth L, Heir T (2012). Bereavement and mental health after sudden and violent losses: a review. Psychiatry.

[31] Mayo Clinic. Complicated grief [Internet]. 2021 Jun 19. Available from: https://www.mayoclinic.org/diseases-conditions/complicated-grief/symptoms-causes/syc-20360374.

[32] Farrugia A, Fraser S, Dwyer R (2017). Assembling the social and political dimensions of take-home naloxone. Contemp Drug Probl.

[33] Fraser S, Farrugia A, Dwyer R (2018). Grievable lives? Death by opioid overdose in Australian newspaper coverage. Int J Drug Policy.

[34] Lankenau S, Wagner K, Silva K, Kecojevic A, Iverson E, McNeely M (2013). Injection drug users trained by overdose prevention programs: responses to witnessed overdoses. J Commun Health.

[35] Wygonik Q, Glassman T, Tucker-Gail K (2021). The role of the social network in fatal opioid overdose prevention: the former opioid user’s perspective. J Drug Issues.

[36] Masferrer L, Garre-Olmo J, Caparros B (2017). Is complicated grief a risk factor for substance use? A comparison of substance-users and normative grievers. Addict Res Theory.

[37] Deo VS, Gilson TP, Kaspar C, Singer ME (2021). The fentanyl phase of the opioid epidemic in Cuyahoga County, Ohio. United States J For Sci.

[38] Beebe J (2014). Rapid qualitative inquiry: a field guide to team-based assessment.

[39] Sangaramoorthy T, Kroenger KA (2020). Rapid ethnographic assessment: a practical approach and toolkit for collaborative community research.

[40] McDonald R, Strang J (2016). Are take-home naloxone programmes effective? Systematic review utilizing application of Bradford Hill criteria. Addiction.

[41] Saldaña J (2015). The coding manual for qualitative researchers.

[42] Guest G, Bunce A, Johnson L (2006). How many interviews are enough? An experiment with data saturation and variability. Field Methods.

[43] Patton MQ (2001). Qualitative research hand evaluation methods.

[44] Bardwell G, Tupper BJ, KW, Kerr T, (2019). “We don’t got that kind of time, man. We’re trying to get high!” Exploring potential use of drug checking technologies among structurally vulnerable people who use drugs. Int J Drug Policy.

[45] Dennis F (2021). Drug fatalities and treatment fatalism: complicating the ageing cohort theory. Sociol Health Illn.

[46] Moore D (2004). Governing street-based injecting drug users: a critique of heroin overdose prevention in Australia. Soc Sci Med.

[47] Benfer I, Zahnow R, Baratt MJ, Maier L, Winstock A, Ferris J (2018). The impact of drug policy liberalization on willingness to seek help for problem drug use. Int J Drug Policy.

[48] Wogen J, Restrepo MT (2020). Human rights, stigma, and substance use. Health Hum Rights.

[49] Kolla G, Strike C (2019). ‘It’s too much, I’m getting really tired of it’: overdose response and structural vulnerabilities among harm reduction workers in community settings. Int J Drug Policy.

[50] Bartoszko A (2018). The lethal burden of survival: making new subjects at risk and the paradoxes of opioid substitution treatment in Norway. Contemp Drug Probl.

[51] Draus PJ, Roddy JK, Greenwald M (2010). A hell of a life: addiction and marginality in post-industrial Detroit. Soc Cult Geogr.

[52] McLean K (2011). The biopolitics of needle exchange in the United States. Crit Public Health.

[53] Snoek A, Fry CL (2015). Lessons in biopolitics and agency: Agamben on addiction. New Bioeth.

[54] Duff C (2009). The drifting city: the role of affect and repair in the development of “enabling environments”. Int J Drug Policy.

[55] Calhoun C, Fassin D, Pandolfi M (2013). The idea of emergency: humanitarian action and global (dis)order. Contemporary states of emergency: the politics of military and humanitarian inventions.

[56] Nichter M, Harthorn BH, Oaks L (2003). Harm reduction: a core concern for medical anthropology. Risk, culture, and health inequality: shifting perceptions of danger and blame.

[57] Garcia A, Das V, Han C (2015). Death as a resource for life. Living and dying in the contemporary world: a compendium.

[58] Vocal New York. Building power and saving lives [Internet]. Available from: vocal-ny.org.

[59] Harm Reduction, Inc. New England Users Union [Internet]. Available from: https://nextdistro.org/resources-collection/new-england-users-union.

[60] Zigon J (2019). A war on people: drug user politics and the ethics of community.

[61] Harm Reduction Works-HRW. Foundations [Internet]. Available from: hrh.414.org/foundationsstart-here-2.

[62] Mendoza S, Hatcher A, Hansen H, Avery J, Avery J (2019). Race, stigma, and addiction. The stigma of addiction.

[63] Kariisa M, Davis NL, Kumar S (2022). Vital signs: drug overdose deaths by selected sociodemographic and social determinants of health characteristics: 25 States and the District of Columbia, 2019–2020. MMWR Morb Mortal Wkly Rep.

[64] Friedman J, Hansen H, Bluthenthal RN, Harawa N, Jordan A, Beletsky L (2021). Growing racial/ethnic disparities in overdose mortality before and during the COVID-19 pandemic in California. Prev Med.

